# Exploring the psychosocial burden of foot complications in diabetes: A cross‐sectional survey and qualitative interview study in a United Kingdom coastal community

**DOI:** 10.1002/jfa2.12038

**Published:** 2024-07-01

**Authors:** Lara S. Chapman, Silva Cochrane, Gill Sykes, Joanne Gill, Jane Nixon, Vijay Jayagopal

**Affiliations:** ^1^ Department of Podiatry Harrogate and District NHS Foundation Trust Scarborough UK; ^2^ Leeds Institute of Rheumatic and Musculoskeletal Medicine University of Leeds Leeds UK; ^3^ Department of Diabetes and Endocrinology York and Scarborough Teaching Hospitals NHS Foundation Trust York UK; ^4^ Leeds Institute of Health Science University of Leeds Leeds UK

**Keywords:** anxiety, Charcot, depression, diabetes, distress, foot, psychosocial, qualitative, survey, ulcer

## Abstract

**Background:**

Foot complications in diabetes are common and destructive, resulting in substantial healthcare costs and high rates of morbidity. Coastal areas have a significantly higher burden of disease. People with diabetes experience disproportionately high rates of psychological health issues, including anxiety, depression and diabetes distress. These can affect self‐management and concordance with preventive measures and treatments of foot complications, negatively impacting on outcomes. Access to psychological health services is variable across the United Kingdom and there is a paucity of high‐quality evidence for the effectiveness of treatments for diabetes distress. This study aimed to explore experiences of psychosocial burden and perceptions and experiences of psychosocial support, among patients with diabetes and foot complications living in a coastal area.

**Methods:**

Patients were eligible to participate if they had experienced diabetes‐related foot complications (amputation, ulceration and/or Charcot neuroarthropathy) within the last 5 years and scored positive for diabetes distress on a validated screening tool (DDS2). Eligible patients completed cross‐sectional questionnaires describing symptoms of diabetes distress (DDS17), anxiety (GAD‐7) and depression (PHQ‐9) and to take part in a face‐to‐face, semi‐structured interview. Questionnaires were analysed using frequencies and interviews were analysed using reflexive thematic analysis.

**Results:**

A total of 183 patients completed the DDS2 screening questionnaire. Of these, 56 (30.6%) screened positive for diabetes distress. Twenty‐seven patients completed DDS17, GAD‐7 and PHQ‐9 questionnaires. Eleven (40.7%) participants indicated high levels of diabetes distress and four (14.8%) indicated moderate distress. Seventeen participants (age range 52–81 years; 12 men) took part in an interview. Four key themes were identified: impact of living with foot problems; emotional consequences of foot problems; experiences and perceptions of psychological support; and strategies to cope with the emotional impact of foot problems.

**Conclusion:**

Diabetes distress was prevalent among patients with diabetes‐related foot complications. Foot problems impacted on participants' daily activities, social lives and ability to work. Despite expressing feelings of ongoing fear, worry and depression relating to their foot problems, only one participant had accessed formal psychological support. Many participants relied on talking to podiatrists at routine appointments and described developing various strategies to cope. The psychosocial burden of living with foot complications in diabetes must not be overlooked by health professionals. Findings from this study can inform the design of future services and interventions.

## 1 INTRODUCTION

Foot complications in diabetes are associated with high rates of morbidity and cost the National Health Service (NHS) approximately £1 billion each year [1]. Complications include diabetic foot ulceration (DFU) and Charcot neuroarthropathy (CN), both of which can result in lower limb amputation. In England, 7957 major diabetic lower limb amputations were reported between 2017/18 and 2019/20 [2]. Coastal areas demonstrate poorer foot health outcomes in diabetes [2], in addition to poorer physical and mental health outcomes in general [3]. This is likely due to a combination of limited employment opportunities, lower paid jobs, higher than average working adult population with low or no educational qualifications, poorer housing and less access to primary and secondary healthcare [3].

People with diabetes experience disproportionately high rates of mental health issues, including anxiety, depression and diabetes distress [4, 5, 6]. Diabetes distress is the emotional response to the burden of living with the condition and managing its demands [7]. Diabetes distress affects one in four people with type 1 diabetes and one in five people with type 2 diabetes [8]. It can affect self‐management and concordance with preventive measures and treatments of foot complications in diabetes, negatively impacting on glycaemic control [9, 10]. Living with foot complications in diabetes can lead to additional emotional distress, particularly due to the reduced mobility and social isolation that often ensues. DFU, CN and lower limb amputations can all lead to low mood and feelings of guilt [11, 12, 13, 14, 15].

There is a paucity of research exploring experiences of psychological support among people with diabetes and foot complications. In a qualitative study involving people with CN, participants described how they could not have managed without emotional support from their family and friends [11]. Similarly, people with diabetes who had undergone minor amputations rely heavily on family support [15]. National guidelines relating to the prevention and management of foot problems in diabetes state that multidisciplinary (MDT) foot care services should have access to psychological support [16]. However, there is no specific advice on what support should be provided and at what timepoint. Access to psychological support for people with diabetes is variable across the United Kingdom (UK); around 75% of patients report they have not been offered this support when they needed it [8]. This issue is amplified by insufficient high‐quality evidence for treatments for diabetes distress to inform clinical practice [17]. Nevertheless, services that have successfully integrated physical and psychosocial healthcare for people with diabetes have indicated a reduction in emotional distress, reduced emergency attendances and unscheduled hospital admissions and improved glycaemic control [18].

This study aimed to explore experiences of psychosocial burden and perceptions and experiences of psychosocial support, among patients with foot complications in diabetes living in a coastal area.

In this study, the term ‘psychosocial’ was defined as the combined influence of psychological factors (e.g., distress, anxiety and depression) and the surrounding social environment (e.g., living in a coastal area, social support and integration) on an individual's well‐being and ability to function [19].

## 2 METHODS

This study involved cross‐sectional surveys to describe participants' symptoms of diabetes distress, anxiety and depression, followed by qualitative individual semi‐structured qualitative interviews. The qualitative interview stage of the study is reported in line with the Consolidated the Criteria for Reporting Qualitative Studies (COREQ) framework [20] (Supporting Information S1). Interviews were developed and analysed from a phenomenological stance to understand the lived experiences of individuals from their own points of view [21].

### 2.1 Ethical considerations

Ethical approval was obtained from the London—Harrow Research Ethics Committee (REC reference 22/PR/0212, May 10, 2022). All participants provided written informed consent. In cases where the researchers had significant concerns about high levels of diabetes distress, anxiety and/or depression indicated or expressed by participants, these concerns were discussed with participants outside of the interview. General Practitioner (GP) input was considered where necessary, and participants were signposted to local and national resources (e.g., local mental health crisis support, Mind and Samaritans). Prior to being interviewed, each participant was informed that the content of the interview was confidential, they could withdraw at any time and their participation or withdrawal would not affect their podiatry care in any way.

### 2.2 Participants

Patients were eligible to participate in the study if they were 18 or over, able to understand and speak English, had a diagnosis of type 1 or type 2 diabetes, had experienced diabetes‐related foot complications (one or more of the following: DFU, lower limb amputation, CN) within the last five years and scored positive for diabetes distress on a validated, 2‐item screening tool, the DDS2 (Table 1). This tool has been used effectively in other diabetes foot services [22] and asks respondents to rate on a six‐point scale the degree to which the two DDS2 items caused distress. An average score of <2 indicates little or no distress, a score between 2 and 2.9 indicates moderate diabetes distress and >3 indicates a high level of diabetes distress. Patients who had undergone lower limb amputation unrelated to diabetes (e.g., trauma‐related), with comorbidities significantly impacting on the lower limb, such as neurological disorders (e.g., history of stroke, multiple sclerosis), or chronic inflammatory joint disorders (e.g., inflammatory arthritis), or with a major cognitive impairment or other impairment that would prevent engagement during the interview, were ineligible to participate. No limitations were applied regarding gender, ethnicity or socioeconomic grouping.

**TABLE 1:** DDS2 screening tool [20].

Item	Not a problem	A slight problem	A moderate problem	A somewhat serious problem	A serious problem	A very serious problem
1. Feeling overwhelmed by the demands of living with diabetes.	1	2	3	4	5	6
2. Feeling that I am often failing with my diabetes routine.	1	2	3	4	5	6

### 2.3 Setting, screening and recruitment

Participants were recruited from one podiatry locality within a single UK NHS Trust site. The locality covers rural and coastal areas. The medical records of patients attending MDT diabetes clinics (podiatry‐led, including vascular consultants, diabetes consultants, orthopaedic consultants and diabetes specialist nurses, but no psychologist) and community ‘high risk’ podiatry clinics were screened. Those with a history of diabetes‐related complications (ascertained from medical records) were screened for diabetes distress using the DDS2 screening tool by diabetes podiatrists. Whilst the DDS2 was not part of previous routine care, podiatrists conducting the screening were accustomed to asking patients about their wellbeing and received training around diabetes distress and the screening tool. Those who scored positive for diabetes distress on DDS2 were deemed eligible and invited, face‐to‐face, to participate in the research study. A convenience sampling technique was employed; all eligible participants were approached. Potential participants were provided with information regarding the aim of the study, the researchers' personal motivations and interests based on their clinical experiences and an overview of what participation would involve.

### 2.4 Data collection

Data collection took place between June 2022 and September 2023. All participants completed three validated, cross‐sectional surveys that are widely used in clinical practice to screen for psychological health issues: DDS17, indicating the severity of diabetes distress symptoms [23, 24], GAD‐7, indicating the severity of anxiety symptoms [25] and PHQ‐9, indicating the severity of depression symptoms [26]. These tools have demonstrated good reliability and validity across different clinical settings [24, 26, 27].

DDS17 uses a six‐point scale for each question, with each item scored from 1 (no distress) to 6 (serious distress) experienced over the past month. Questions relate to four subscales targeting different areas of potential diabetes‐specific distress: interpersonal distress (not receiving understanding and appropriate support from others), regimen distress (concerns about diet, physical activity and medications), physician distress (worries about access, trust and care) and emotional burden (feeling overwhelmed by diabetes). The total mean score is calculated by summing up responses to all items and dividing by 17. A score of ≥3 indicates high distress; 2.0–2.9 indicates moderate distress and a score of ≤2 indicates minor or no distress. GAD‐7 uses a seven‐point scale, with each item scored from 0 (not at all) to 3 (nearly every day), relating to symptoms of anxiety over the past two weeks, adding up to a possible total of 21. Scores of 5, 10 and 15 are cut‐off points for mild, moderate and severe anxiety, respectively. Similarly, PHQ‐9 uses a nine‐point scale and classifies symptoms of depression over the past two weeks on a scale of 0 (not at all) to 3 (nearly every day), with a maximum score of 27. Scores of 5, 10, 15 and 20 are cut‐off points for no depression, mild, moderate, moderately severe and severe depression, respectively.

All participants who completed the surveys were asked to indicate if they were willing to participate in an individual, face‐to‐face semi‐structured interview. An interview topic guide (Supporting Information S1) was developed by the full research team and was based on previous literature, clinical experience (incorporating the perspectives of podiatrists, a diabetes consultant and a diabetes specialist nurse) and feedback from patient and public involvement (PPI) contributors. The topic guide was reviewed iteratively throughout the interview process, with additional questions of interest added as appropriate, based on findings from previous interviews.

Interviews were conducted jointly by two researchers (GS and SC—both female, MSc, and advanced specialist diabetes podiatrists with previous clinical research experience) or by one of the two researchers. Both interviewers were known to participants and the interviewers explained their personal motivations for doing the research (to understand the impact of foot problems and to improve psychosocial support for patients with diabetes). Interviews were conducted in participants' own homes or within a community podiatry centre, depending on each participant's preference, and were audio recorded using a digital voice recorder. Participants attended the interview alone, with the exception of one participant who attended with his wife. The interview duration ranged from 16 to 48 min. Field notes were made during the interview when two researchers were present and immediately after the interview when one researcher was present. Both researchers kept a reflexive diary throughout the data collection period, documenting personal reflections of their values, interests and insights in relation to each interview and debriefed with each other after every interview.

### 2.5 Analysis

Survey data were analysed using frequencies (number (*n*) and %). Qualitative data was analysed using reflexive thematic analysis to identify and explore themes in relation to the research aim. This was achieved by grouping together similar codes to understand participants' collective experiences and identify similarities and differences between participants' perspectives [28]. Interviews were transcribed verbatim and read and re‐read by one researcher (LSC) to achieve familiarisation with the dataset. Transcript data were then inputted into Microsoft Excel (Microsoft, 2022) and coded line‐by‐line by the researcher. Coding decisions were discussed by three researchers (LSC, SC and GS) until consensus was reached, and similar codes were grouped into themes. All themes were derived from the data; none were identified in advance. Emerging results, and the content of themes, were discussed with the wider research team over three debriefing meetings during the analysis process. Each theme is supported by verbatim quotations to enhance conformability [29]. We did not employ member checking in this study due to its potential to raise ethical issues [30].

### 2.6 Patient and public involvement

PPI contributors from a local NHS Trust PPI group reviewed all patient‐facing documents and the interview topic guide. Suggested changes relating to the wording of interview questions (to improve readability) were incorporated into the final documents.

## 3 RESULTS

### 3.1 Participant demographics

A total of 183 patients met the clinical eligibility criteria and completed the DDS2 screening survey. Of these, 56 (30.6%) screened positive for diabetes distress and were invited to participate in the full study. Out of 56 invited to participate in the study, twenty‐seven patients consented and completed DDS17, GAD‐7 and PHQ‐9 surveys. Survey participant demographics are presented in Table 2.

**TABLE 2:** Survey participant demographics.

	Participants (*n* = 27)
Age	Range 48–79, mean 65.37 (SD 9.34)
Ethnicity	27 (100%) White British
Sex	21 (77.8%) men, 6 (22.2%) women
Highest education level	17 (63%) high school, 5 (18.5%) A‐levels, 5 (18.5%) degree level
Work status	4 (14.8%) part time, 2 (7.4%) full time, 12 (44.4%) retired, 9 (33.3%) unemployed
Income	10 (37%) less than £10,000 per annum, 8 (29.6%) £10,000–£19,999 per annum, 4 (14.8%) £20,000–£29,999, 1 (3.7%) £30,000–£39,999, 1 (3.7%) over £50,000, 3 (11.1%) prefer not to say

### 3.2 Cross‐sectional survey results

#### 3.2.1 Diabetes distress (DDS17)

Eleven (40.7%) participants indicated high levels of diabetes distress, four (14.8%) indicated moderate distress and the remaining 12 (44.4%) indicated no or minor distress.

#### 3.2.2 Anxiety (GAD‐7)

Six (22.2%) participants indicated symptoms of severe anxiety, six (22.2%) indicated symptoms of moderate anxiety, four (14.8%) indicated symptoms of mild anxiety and the remaining 11 (40.7%) indicated no anxiety symptoms.

#### 3.2.3 Depression (PHQ‐9)

Six (22.2%) participants indicated symptoms of severe depression, one (3.7%) indicated symptoms of moderately severe depression, nine (33.3%) indicated symptoms of moderate depression, three (11.1%) indicated symptoms of mild depression and the remaining eight (29.6%) indicated no depression symptoms.

### 3.3 Semi‐structured interviews

All 27 participants who completed surveys indicated that they were willing to take part in an interview. Seventeen participants completed an interview (age range 52–81 years, mean [SD] 64 [8.32]). Interview participant demographics are presented in Table 3. The remaining 10 participants either declined participation at the recruitment follow‐up stage due to declining health or were unable to attend the interview in the recruitment timeframe due to other commitments.

**TABLE 3:** Interview participant demographics.

Participant ID	Sex	Foot complication(s)	DDS17 score	Severity of diabetes distress	GAD‐7 score	Anxiety severity	PHQ‐9 score	Depression severity
P1	M	Current DFU	1.4	Minor	1	None	1	None
P2	F	Current DFU	4.5	Severe	17	Severe	22	Severe
P3	M	Previous amputation	2.4	Moderate	4	None	11	Moderate
P4	F	Previous amputation, current CN	3.8	Severe	15	Severe	15	Moderately severe
P5	M	Previous amputation, previous CN	3.3	Severe	12	Moderate	14	Moderate
P6	M	Previous amputation	2.5	Moderate	16	Severe	21	Severe
P7	M	Current DFU	1.3	Minor	17	Severe	20	Severe
P8	M	Current DFU	3.6	Severe	13	Moderate	14	Moderate
P9	F	Current DFU	1.5	Mild	1	None	4	None
P10	F	Current DFU, previous CN	1.7	Minor	1	None	9	Mild
P11	F	Previous amputation	1.1	Minor	3	None	6	Mild
P12	F	Current DFU	1.9	Minor	7	Mild	10	Moderate
P13	M	Previous amputation, current DFU	2.1	Moderate	10	Moderate	10	Moderate
P14	M	Current ulcerations	3	Severe	0	None	6	Mild
P15	M	Previous amputations, current DFU	3.2	Severe	15	Severe	19	Severe
P16	M	Previous amputation, current DFU	1.3	Minor	11	Moderate	11	Moderate
P17	M	Previous amputation, current CN and DFU	3.3	Severe	13	Moderate	12	Moderate

Four key themes were identified: impact of living with foot problems; emotional consequences of foot problems; experiences and perceptions of psychological support; and strategies to cope with the emotional impact of foot problems.

#### 3.3.1 Theme 1—Impact of living with foot problems

Participants described the impact of living with foot problems on their everyday activities and hobbies, including walking, shopping, gardening and driving:

It was difficult in some respects – I had the dogs to walk, I couldn’t do that. I can’t drive … I haven’t shopped for five years.

P10

Why volunteer to do something? I stopped all that, I can’t sit on the tiny chairs anymore, because I can’t get out. I’ve done it, I’ve had a go.

P3

For some participants, the impact of not being able to walk or drive affected their friendships and social participation:

I usually go to the quiz at the pub on a Wednesday night. I couldn’t go when my foot was bad. For quite a while I didn’t go.

P13

Participants also described the impact of their foot problems on employment. Most participants had made changes at work, including changing their job role, reducing their hours or giving up work entirely. Most participants had worked or were currently working in manual jobs, including catering, bar work, caretaking, building and farming:

I tried at the beginning when I had the first ulcer under me toe. I tried to work to a certain extent then, but after the toe amputated I went back to work, it wasn’t successful and I ended up with Charcot foot and pretty much not been able to do a lot since, because I’m in the building trade, it’s not good for going out.

P17

I was [working] ‘til I went into hospital, I’ve been off a couple of years now on the universal credit. I used to work for a mate who has got a fruit and veg business and I’d be driving about – jumping on and off. So quite manual really, which I have been all my life. But yeh when it suddenly kind of stops. I thought ah things will come right soon, I’ll be back to work, but it didn’t quite work out like that. Probably wishful thinking really.

P5

Participants who were unable to work expressed a desire to work again and highlighted the negative financial impact of not working:

I don’t want to lose any more [toes]. I want the ability to walk and work.

P14

Just not working. You’ve got to change your complete – from always having money to having no money, it’s bloody hard. We’ve now got a bedroom, house full of Christmas presents, so all kids – you can’t just go out and spend a fortune like you could before.

P4

Other participants were unwilling to give up work, despite their foot problems and advice from health professionals to stay off their feet as much as possible:

Very hard really like, obviously in a farming environment you can’t really keep off your feet and keeping it clean is quite hard – you never know if you’ll get something down your welly, muck in and that like, like I say it is kind of stressful really.

P8

Participants' finances were also affected by having to pay others to transport them and needing to buy different footwear:

If I buy any boots, they’re expensive. I went to Clarks ‐ £80! I wore them twice, they were tight on me. I gave them away – I’ve given five pairs of boots away. They weren’t cheap boots.

P7

Participants highlighted how their foot problems not only impacted their own activities but also those of their family members; for example, relying on others to do more physical work around the house. For some participants, this created feelings of guilt and a loss of self‐worth.

#### 3.3.2 Theme 2—Emotional consequences of foot problems

Participants described a range of emotions as a result of their foot problems, including being shocked, worried and fed up. Theme two is closely linked to Theme 1, with impact on physical and social activities and work and finances having psychological consequences for participants:

I found not being able to walk and get out does make your life a little less fulfilled in a lot of ways.

P6

I’m fed up. I’m not trying to be funny, I’m just fed up. She [wife] goes out, bus stops over there, but I feel awful because I can’t go with her. I used to go out on the bus with her but it’s awkward getting on and off the bus. My problem is steps.

P7

I had trouble sleeping. Worry with my foot and the money and everything else.

P13

The negative impact of foot complications in diabetes on psychological health was exacerbated among participants who lived alone and felt socially isolated.

Other participants emphasised the psychological impact of not being able to exercise, as well as having to wear ongoing dressings for ulcers:

But yeh – I thought I was doing alright, exercising and everything else, it’s hard. It’s psychological – your mind is telling you you should be exercising but then you’ve got to accept it. I was quite into my fitness, gym twice a week, a lot of walking.

P5

I am over clean – I couldn’t really wash my feet properly, that got me down mentally. Not being able to have a proper shower, with a boot on – having to do in between my toes with wet wipes. It was the boot that really got me. One time I had dressings on both feet – I was walking around with two boots on.

P2

Some participants highlighted the emotional impact of how suddenly foot complications could start or deteriorate, particularly in cases where they had been hospitalised:

The decision was made at the hospital to have my toe removed – that was on the Weds – I went into hospital on the Thurs for the second toe on my right foot, I was released from the hospital on the Saturday so it was… a bit of a shock at the time, as you can imagine, that it was going to happen.

P6

A lot happened in a short space of time and it was difficult to cope with at the time.

P1

Participants described fear and panic resulting from their foot complications, emotions that persist even during times when there was no active problem:

It’s in your mind all the time … I panic. Like ‘oh my foot’s going to come off’, you know what can happen with diabetes, all the stories and that. It does sort of depress you like, to be honest, I had a big one and I went to see them – it was frightening, it was that big, I thought this is the end, like? It frightened me, like.

P8

I’m more conscious of them than I was before. This one it’s swelling up like it did before. So I’m a bit nervous this time. It might be infected or something.

P10

Additionally, participants described feeling guilty about the impact of their foot problems on their family members:

I’ve affected [my wife], I’ve affected my children, I’ve affected them emotionally … I’ve affected them mentally in the case of creating anxieties because of being hospitalised. Worrying about me. Suddenly my wife is having to do much more of the physical work. [I] feel that I’ve been incredibly selfish with my diabetes.

P15

When I got sent from *[name of hospital]* hospital to *[name of hospital]* hospital it was a bit more difficult – that upset her *[participant’s sister]*. I was in there for seven weeks. When I eventually got out and went home she wasn’t well at all.

P10

#### 3.3.3 Theme 3—Experiences and perceptions of psychological support

Only one participant had accessed formal psychological support in relation to their foot problems in diabetes. Two others had been offered support but declined:

I was a bit stressed at time, I did feel a bit down, I wasn’t sort of right, and she tried to get me to talk to somebody and I sort of refused. I maybe should have done really but … you get old and think ‘I don’t really need to talk to people’.

P8

Some participants perceived that accessing formal psychological support would be difficult:

Mental health? Well. I did used to go to [name of mental health worker], it was sort of one on one cognitive therapy but I mean nowadays if you need it you’ve got no chance.

P1

I mean they gave me a number to ring if I asked. But it’s probably a two year waiting list before you get to speak to anyone … I’ve ended up sorting myself out.

P12

Participants varied in their perceptions about the benefits of formal psychological support. Some acknowledged that talking to someone might help them feel better, whilst questioning the overall impact this would have on their foot complications:

Nothing’s going to change the actual outcome of things. It might just make things a bit more bearable, talking to somebody … yeh, probably good to talk to somebody, but whether it would change anything, I don’t know. I could tell somebody but what’s… it’s only telling them that’s going to make me feel any better, isn’t it, it’s what they’re going to do?

P17

I don’t think it would have made any difference really.

P16

When questioned about their emotions, psychological support and coping strategies, some participants, all of whom were men, rapidly redirected the conversation to physical support. Others explicitly reflected on their personality type, identifying that formal psychological support did not align with their self‐perception:

Oh you don’t need it [empathy and sympathy]. I don’t need it. No … I don’t really want it … I’ll come to all my appointments and everything but I aren’t the type of person.

P4

I’m not a support fellow. We just get on with it … I don’t like bothering doctor, he’s a smashing bloke.

P7

#### 3.3.4 Theme 4—Strategies to cope with the emotional impact of foot problems

In lieu of formal psychological support, participants described seeking social support from friends, family, neighbours and their wider communities:

I just talk to people on the phone. Well I’ve got a lot of good friends, relations, they ring up to try to keep you going.

P6

I think if you have good friends, and you have a good connection, like I have my church for instance. So I’ve got loads of people I can call on, and they will come if I need it, they will come to my assistance, they will listen to my woes and my… crying and give me a hug, and they go away and I’ll feel ten times better.

P11

Most participants described how they sought emotional support from podiatrists at routine appointments and wanted to see the same podiatrist every time for continuity of emotional support. Participants highlighted the importance of building trust, and talking to podiatrists at their foot health appointments was perceived to have a positive impact on psychological health:

Well I trust you enough. To be fair it creates another issue to have to go and see somebody. I’d rather have my foot looked after and get right and I can deal with myself as it is. Talking to you lot about it is good. I don’t think you need anybody else. Yeh, yeh, you’ve got my trust.

P6

There are people – I used to know the vicar and priest here, I don’t need that. I know some people do need that. I know it sounds stupid. The people that I’ve got top of my list – it’s you lot [podiatrists]. Seriously!

P3

When discussing their psychological health, many participants described ‘getting on with it’ and ‘keeping going … like anybody would’:

I just get on with it. I have tried talking about it. Most of the family’s far away anyway. It seems the impression I get is that they all have their own problems to deal with. I have no choice but to get on with it.

P16

You just have to live with it, haven’t you.

P1

You have no choice, you’ve got to deal with it and get on with it regardless, whether it’s one foot or both feet, you’ve got to get on with it. You’ve got to crack on, haven’t you.

P17

Some participants actively tried to take a positive attitude in relation to their health:

It is what it is. I try to be positive … Me life’s changed since I’ve had the amputation – obviously, I mean you see it all every day. Things go through my mind – but I always think you know it could have been worse, a lot worse. It’s a matter of getting on with life and be happy … but it certainly affects things.

P5

Other participants compared themselves to other people who they perceived to be worse off, often feeling ‘luckier’ than others:

I try not to let it bother me – there’s people out there with far more horrible conditions that what I’ve got.

P14

It has got quite depressing, I’ll be honest. But look, you’ve just got to crack on with it – there’s worse people, you see people in hospital – I went in and one fellow had gone in for two toes off and he never came back, he died on the table. So you can see everything around it – and you just think, get on with it.

P6

One participant described his foot problems as ‘tiny’, when comparing his health to others:

The last thing I want to do is tell her I have these tiny problems with me.

P3

Some participants revealed the benefits of having tasks to focus on; for example, painting the house or walking the dog. One participant described writing in a diary to deal with her worries:

I talk to myself about things. I write a diary. I find that really good because that’s a way of putting it down. And then it’s gone. Yeh, if I’m a bit down, which is unusual for me but if I do feel a little bit – what’s wrong now, what’s wrong now – I write that down. Then it’s out of my mind, I stop worrying about it.

P10

## 4 DISCUSSION

This study increases our understanding of experiences of psychosocial burden and the perceptions and experiences of psychosocial support, among podiatry patients with foot complications in diabetes living in a coastal area. In congruence with previous research in this area, our findings indicated that DFU, CN and lower limb amputations impacted participants' daily activities, social lives and ability to work and had negative consequences on psychological health [6, 11, 13, 14]. These findings were supported by data from cross‐sectional DDS17, GAD‐7 and PHQ‐9 surveys, indicating a high prevalence of symptoms relating to diabetes distress, anxiety and depression, respectively.

Despite expressing feelings of ongoing fear, worry and depression relating to their foot complications, only three interview participants recalled being offered formal psychological support, and only one had accessed this, highlighting the divide between physical and mental health services recently reported by Diabetes UK [8]. However, our findings highlight different attitudes towards formal psychological support. Some participants readily discussed the emotional impact of their foot complications but did not identify as being the type of people who needed formal support, for example, support from a GP or psychologist. In line with previous research, participants highlighted the support they had received from their friends and family. Additionally, many participants revealed that they relied on podiatrists for support related to the emotional impact of their foot complications, with some participants in our study perceiving they did not need to talk to anybody else about their feelings. The severe pressures currently faced by the NHS following years of chronic under‐resourcing are well documented, with many staff members experiencing burnout and significant numbers intending to leave their jobs [31]. Workforce retention is a key priority for NHS England with coastal areas consistently struggling to recruit and retain staff [3]. In NHS podiatry specifically, staff retention and subsequent understaffing are ongoing issues [32]. Podiatrists working in this area typically see patients over a long period of time, whilst participants in our study indicated the importance of seeing the same podiatrist at each visit. The subsequent impact on podiatrists, the extent of support in place for podiatrists to be able to help patients who need emotional support and the type of support or training that could be effective are areas for future research. For example, the use of motivational interviewing in diabetes podiatry is increasing [33, 34, 35], and in the absence of formal psychology input in the diabetes foot MDT, training podiatrists in motivational interviewing may be beneficial. Additionally, training around the Making Every Contact Count approach, which encourages health professionals to use the opportunities arising during routine interactions with patients to have conversations about making positive improvements to health [36], could support podiatrists to approach emotional wellbeing with patients.

To our knowledge, this is the first qualitative study to explore the experiences and attitudes towards psychological support in patients with foot complications in diabetes and provides a grounding for future research. Findings from our study must be considered in light of limitations, however. Firstly, participants were recruited directly from podiatry clinics and were known to the interviewers, which may have introduced bias during data collection, and participants may have been discouraged from talking honestly about their clinical care or may have otherwise modified their responses as a result of knowing the researchers' motivations [37] However, our experience was that existing trust between the interviewers and participants facilitated an open discussion about the sensitive topic matters in the interview. Additionally, all interviews were initially analysed independently by a member of the research team who was not involved in the clinical care of participants. Our study was limited to a single NHS Trust; thus, our findings may not reflect the experiences and attitudes of patients in other areas due to variations in service provision, particularly areas where psychologists are successfully embedded in the diabetes/podiatry MDT. Whilst our study captured a broad range of patients regarding gender, age, type and duration of foot problems and diabetes distress level, all participants were White British and we excluded patients unable to speak and understand English to ensure that participants could participate fully in the interviews. Therefore, our findings may not be transferable to different cultures and ethnicities. Additionally, our findings may not reflect the views of patients with foot complications in diabetes who are less willing to participate in research, and we acknowledge that the characteristics of participants who declined to take part in this study may have been different to those who completed the surveys and participated in an interview. The potential for recall bias must also be taken into consideration, given that our eligibility criteria included participants who had experienced foot complications within the last 5 years [38]. Furthermore, our study focused exclusively on patients with foot complications; exploration of psychosocial burden and support for patients with diabetes who have not experienced DFU, amputation or CN, with a focus on prevention, is an important area of further study. Future research could also explore the views of patients with diabetes who are not experiencing any diabetes distress to explore how they are coping and establish any psychosocial support they may have accessed.

Our findings could inform the design of psychosocial interventions for patients with foot complications in diabetes and of future services integrating psychosocial support into clinical practice. The emotional impact of living with foot complications in diabetes is clear and should not be overlooked by health professionals. However, patients may have differing psychosocial needs and coping strategies, and not all patients may benefit from accessing formal psychological support. Podiatrists who frequently treat the same patients with foot complications in diabetes appear to be highly trusted and may be utilised by patients for more informal psychological support. Future research should also focus on the subsequent impact on podiatrists and how they can be best supported to address patients' psychosocial burden.

## 5 CONCLUSIONS

In this study, diabetes distress was prevalent among participants with foot complications living in a coastal area. Foot complications impacted participants' lives, including their daily activities, social lives and ability to work. Despite expressing feelings of ongoing fear, worry and depression relating to their foot problems, only two participants had accessed formal psychological support. Participants described various coping strategies, with many relying on talking to their podiatrists about the emotional impact of their condition at routine appointments. Findings from this study can inform the design of future interventions and services that integrate psychosocial support.

## AUTHOR CONTRIBUTIONS

**Lara S. Chapman**: Conceptualization; data curation; formal analysis; funding acquisition; investigation; methodology; supervision; visualization; writing – original draft. **Silva Cochrane**: Investigation; project administration; formal analysis; writing – review & editing. **Gill Sykes**: Investigation; project administration; formal analysis; writing – review & editing. **Joanne Gill**: Methodology; writing – review & editing. **Jane Nixon**: Methodology; writing – review & editing. **Vijay Jayagopal**: Conceptualization; funding acquisition; methodology; supervision; visualization; writing ‐ review & editing.

## CONFLICT OF INTEREST STATEMENT

The authors declare no conflicts of interest.

## ETHICS STATEMENT

This study received a favourable opinion from the London—Harrow Research Ethics Committee (REC reference 22/PR/0212). All participants provided written informed consent prior to participation.

## CONSENT FOR PUBLICATION

Not applicable.

## Supporting information

Supporting Information S1

## Data Availability

The datasets generated and/or analysed during the current study are not publicly available to ensure that participants' anonymity is maintained.
